# AI-enhanced 3D tooth movement forecasting in clear aligner therapy using deep morphometric modelling: A prospective validation study

**DOI:** 10.6026/973206300214753

**Published:** 2025-11-15

**Authors:** Rattan Khurana, Archana, Kanish Aggarwal, Sharvari Bhat, Afrah Fatima, Abida Parveen, Heena Dixit

**Affiliations:** 1 Department of Orthodontics and Dentofacial Orthopaedics, Gian Sagar Dental College and Hospital, Ram Nagar (Rajpura), Patiala, Punjab, India; 2 Department of Orthodontics and dentofacial orthopedic, HKDETS dental college and hospital humnabad, Bidar, Karnataka, India; 3 Department of Orthodontics, Bhojia Dental College and Hospital, Baddi, Himachal Pradesh, India; 4 Consultant Orthodontist, the Dental Residence, Pune, Maharashtra, India; 5 Department of Orthodontics, Panineeya Institute of Dental Sciences and Research Centre, Hyderabad, Telangana, India; 6 Department of Orthodontics and Dentofacial Orthopaedics, MMCDSR, Maharishi Markandeshwar (Deemed-to-be University), Mullana, Ambala, Haryana, India; 7 Department of Dentistry, Sheikh Bhikhari Medical College, Hazaribagh, Jharkhand, India; 8 Department of Medical Health Administration, Index Institute, Malwanchal University, Index City, Nemawar Road, Indore, Madhya Pradesh, India

**Keywords:** Orthodontic aligners, artificial intelligence, clinical decision-making, treatment outcome prediction, orthodontics

## Abstract

Clear aligner therapy often encounters early tracking deviations that compromise treatment efficiency, creating a need for predictive tools that identify risk at the outset. Therefore, it is of interest to develop and validate a deep morphometric AI model capable of forecasting early aligner tracking deviation using baseline and first-week 3D intraoral scans. Hence, a prospective sample of 40 adults was analyzed using a graph-convolutional neural network trained on geometric mesh features extracted from sequential scans. The model demonstrated strong performance, achieving 85% accuracy with an RMSE of 0.19 mm in predicting clinically significant early deviation. Thus, we show that AI-driven morphometric analysis offers a promising approach for early risk detection and improved treatment planning in clear aligner therapy.

## Background:

Clear aligner therapy (CAT) relies on precise biomechanical execution, yet deviations between planned and actual tooth movement-termed tracking failure-remain a major clinical challenge. Recent evidence shows that 27-45% of aligner cases experience clinically relevant tracking discrepancies, often necessitating unplanned refinements or auxiliary interventions [1]. Although digital treatment planning software provides idealized simulations, these platforms lack predictive capability for patient-specific movement accuracy [2]. Advances in deep learning and 3D morphometric modelling enable extraction of complex geometric features from intraoral scans, providing new opportunities to quantify and predict early deviations in aligner tracking [3]. Neural network models have demonstrated strong performance in forecasting orthodontic tooth movement, achieving sub-millimeter predictive accuracy in controlled experimental conditions [4]. Furthermore, AI-based systems integrating 3D surface correspondence have shown utility in evaluating aligner fit dynamics and occlusal changes during treatment progression [5]. However, no prospective clinical study has validated an AI model designed to predict aligner tracking failure based exclusively on baseline and first-week 3D scan morphometry. Therefore, it is of interest to develop and validate a deep morphometric prediction model to identify patients at risk of early aligner tracking deviation, supporting more individualized and efficient orthodontic management.

## Materials and Methods:

This prospective observational study was conducted at a university orthodontic clinic following institutional ethics approval. Forty adult patients initiating clear aligner therapy were consecutively recruited based on inclusion criteria of permanent dentition, mild-to-moderate malocclusion, and availability of pre-treatment and first-week intraoral scans. Exclusion criteria included periodontal disease, craniofacial anomalies, previous orthodontic therapy, or inconsistent aligner wear. Baseline digital models (T0) and first-week scans (T1) were captured using a standardized intraoral scanner (accuracy ≤20 µm). A deep morphometric prediction model employing graph-convolutional neural network architecture was trained on 3D mesh features extracted from T0 and T1 scans. The primary outcome was early aligner tracking deviation, defined as >0.25 mm discrepancy between predicted and actual tooth positions at week four. Model performance was evaluated using accuracy, sensitivity, specificity, and root-mean-square error (RMSE). Statistical analyses were performed using Python and SPSS v27, with significance set at p<0.05.

## Results:

In total, 40 patients were analyzed, with mean baseline displacement magnitude of 0.82 ± 0.21 mm across monitored teeth. First-week aligner deviation averaged 0.18 ± 0.07 mm, indicating generally good initial tracking. However, 30% of patients showed early deviation exceeding 0.25 mm at one or more tooth sites, predominantly in canines and premolars. Overall, baseline morphometric variability and early deviation values demonstrated sufficient heterogeneity to train and validate the prediction model (Table 1 - see PDF). The AI model correctly predicted early aligner tracking deviation in 85% of patients, demonstrating strong clinical utility. Sensitivity was 81%, indicating reliable identification of patients truly at risk for early tracking failure. Specificity reached 88%, meaning false alerts were minimal and prediction stability was high. The overall RMSE of 0.19 mm reflected high precision in forecasting early tooth movement relative to actual clinical outcomes (Table 2 - see PDF).

## Discussion:

The present study demonstrates that a deep morphometric model derived from early 3D intraoral scan analytics can effectively predict aligner tracking deviation with high accuracy. Our findings align with recent evidence showing that geometric deep-learning approaches are capable of detecting subtle morphological variations that influence orthodontic tooth movement precision [6]. Early tracking deviation remains a major contributor to unplanned refinements, and its prediction has been emphasized as essential for improving clinical efficiency and reducing treatment burden [7, 8]. Previous investigations have shown that baseline shape variability and tooth-specific biomechanical responses are significant determinants of aligner predictability, supporting the relevance of incorporating 3D mesh-based inputs into AI models [9]. The model's accuracy of 85% and RMSE of 0.19 mm are comparable to other AI-driven orthodontic prediction systems that have reported sub-millimeter precision in simulated and clinical environments [10, 11]. Notably, our sensitivity and specificity values exceeded those documented for earlier machine-learning models focused on refinement risk or aligner fit assessment, suggesting that morphometric learning captures clinically meaningful deformation patterns [12]. Integration of early treatment scans (T1) also appears advantageous, as prior work indicates that week-one biomechanics strongly correlate with long-term tooth movement trajectories [13]. Clinically, early identification of high-risk patients may enable targeted monitoring, optimized staging, and timely implementation of auxiliaries, thereby improving treatment outcomes. However, the study is limited by its modest sample size and single-center design. Future multicenter datasets and incorporation of force-based metrics may further enhance predictive robustness [14, 15].

## Conclusion:

The deep morphometric prediction model demonstrated strong accuracy and reliability in identifying early aligner tracking deviations, highlighting its potential clinical value. The system effectively detected subtle biomechanical discrepancies that precede treatment inefficiencies by leveraging baseline and first-week 3D scan features. Thus, we show the integration of AI-driven morphometric analytics into routine aligner monitoring to enhance precision, reduce unplanned refinements, and optimize patient outcomes.
